# Clinical measures associated with aspiration risk in multiple system atrophy: a cross-sectional study

**DOI:** 10.1016/j.prdoa.2025.100401

**Published:** 2025-10-17

**Authors:** Jun Tanimura, Toshiyuki Yamamoto, Yuji Takahashi

**Affiliations:** a Department of Neurology, National Center Hospital, National Center of Neurology and Psychiatry (NCNP), 4-1-1 Ogawa Higashicho, Kodaira, Tokyo 187-8502, Japan; b Department of Neurology, Aizawa Hospital, 2-5-1 Honjo, Matsumoto, Nagano 390-0814, Japan

**Keywords:** Multiple system atrophy, Aspiration, Videofluoroscopic swallowing examinations, Dopamine transporter imaging, Barthel index

## Abstract

**Introduction:**

Identifying clinical measures associated with aspiration risk is crucial for guiding clinical decisions in patients with multiple system atrophy (MSA). We investigated whether basic clinical measures—including the Barthel index (BI), Unified MSA Rating Scale (UMSARS), and dopamine transporter single-photon emission computed tomography imaging (DaT imaging)—are associated with aspiration risk in patients with MSA.

**Methods:**

This cross-sectional, single-center study analyzed patients with MSA who underwent videofluoroscopic swallowing examinations (VF) and DaT imaging within 12 months of each other. Clinical measures were assessed within 3 months of VF. Patients were categorized into aspiration and non-aspiration groups based on VF findings. Multivariable modified Poisson regression and receiver operating characteristic (ROC) analyses were performed to explore the associations between clinical measures and aspiration, and their discriminative ability.

**Results:**

Among 105 patients with MSA (58 females; median age: 63 years [range: 43–87 years]), 28 (26.7 %) showed aspiration on VF. The aspiration group showed significantly lower BI and higher UMSARS scores than the non-aspiration group. In multivariable regression analyses, BI was consistently and significantly associated with aspiration across all models. ROC analysis demonstrated moderate discriminative ability both BI (AUC 0.77, 95 %CI 0.66–0.87) and UMSARS (AUC 0.73, 95 %CI 0.63–0.83) for aspiration, with overlapping confidence intervals. The association was particularly notable for the parkinsonian MSA subtype.

**Conclusion:**

The BI shows potential as a clinical marker of aspiration risk in MSA, particularly in the parkinsonian subtype, which may help guide the timing of swallowing assessments even in the absence of overt dysphagia symptoms.

## 1 Introduction

Aspiration is a critical complication of multiple system atrophy (MSA) that significantly affects the prognosis through malnutrition, aspiration pneumonia, and sudden death [1]. As a milestone of severe dysphagia, aspiration often guides clinical decisions regarding compensatory feeding strategies or aspiration prevention surgery [2]. While videofluoroscopic (VF) and videoendoscopic (VE) swallowing studies remain the gold standard for assessing aspiration risk, they require specialized equipment and expertise, making them impractical for routine screening. Identifying reliable clinical markers using readily available clinical information may help optimize the timing of detailed assessments.

Although subjective questionnaires have shown value in identifying dysphagia in MSA [3], objective markers of aspiration risk remain underexplored. The Barthel index (BI) [4] has been shown to be associated with dysphagia [5], but its association with aspiration risk and discriminative ability remains unexplored. Similarly, although the Unified Multiple System Atrophy Rating Scale (UMSARS) [6] comprehensively assesses disease severity, its utility as an aspiration risk marker remains unknown. A recent study on MSA supports an association between swallowing impairment severity and dopamine transporter single-photon emission computed tomography (SPECT) imaging (DaT imaging) findings [7], though its specific relationship with aspiration risk and discriminative ability remains untested.

We investigated whether BI, UMSARS, and DaT imaging are associated with aspiration risk in MSA and assessed their discriminative ability.

## 2 Methods

### 2.1 Study design and setting

This cross-sectional, single-center study reviewed all consecutive patients diagnosed with clinically established or probable MSA according to the International Parkinson and Movement Disorder Society (MDS) MSA criteria [8] at our center between November 2015 and December 2023. The inclusion of probable MSA cases was supported by their recently validated high diagnostic accuracy [9]. Based on the predominant motor phenotype, patients were classified into MSA-cerebellar (MSA-C) or MSA-parkinsonian type (MSA-P) [8]. VF was performed as part of our center's standard protocol, regardless of suspected dysphagia.

Inclusion criteria were patients who underwent DaT imaging within 12 months before or after VF. The exclusion criteria were patients with a nasogastric tube or gastrostomy, as they were unsuitable for a study aimed at early markers of dysphagia, and those without predefined clinical assessments described below within 3 months of their VF to ensure the quality of the clinical assessment.

### 2.2 Clinical assessments

Clinical measures assessed within 3 months of the VF included demographic characteristics (age, sex, and body mass index [BMI]) and disease status (MSA subtype, disease duration, Mini-Mental State Examination [MMSE] [10], BI, UMSARS, and mean specific binding ratio [SBR] obtained from DaT imaging). The BI evaluates the activities of daily living (ranging from 0 to 100, with higher scores indicating a better functional status).

### 2.3 Videofluoroscopic swallowing examinations (VF)

VF was performed as described previously [11]. Patients with dysphagia initially underwent testing with 5 mL of a thickened liquid. If no aspiration was observed, additional tests were conducted using 5 and 10 mL of thinner liquids. Aspiration was defined as liquid entering the trachea beyond the vocal cords (penetration-aspiration scale score 6–8). Patients were categorized into aspiration and non-aspiration groups based on VF findings. The VF evaluations were conducted independently of other clinical and imaging assessments.

### 2.4 DaT imaging

DaT imaging using [^123^I]FP-CIT (DaTSCAN; Nihon Medi-Physics, Tokyo, Japan) was performed and analyzed with DaT View software (version 8.0; Nihon Medi-Physics) using the Southampton method, as described previously [11]. SBR was defined as the ratio of striatal binding to binding in a reference region. Striatal volumes of interest (VOIs) were manually adjusted based on cross-sectional references. The reference VOI was placed in the brain parenchyma using an *iso*-contour threshold of 30 %. The average of the right and left SBRs (“SBR*mean*”) was used for analysis.

### 2.5 Statistical analysis

Descriptive statistics are presented as median and interquartile range (IQR) for continuous data or counts and proportions for categorical data. Missing MMSE scores (n = 5) were imputed using the mean. Group comparisons were performed using two-tailed Mann–Whitney *U* test for continuous variables and two-tailed Fisher’s exact test for categorical variables, with Bonferroni correction for multiple comparison. Variables with corrected p-values < 0.05 (BI and UMSARS) were selected to investigate their association and discriminative ability.

We assessed multicollinearity prior to regression analysis using Spearman's correlation coefficients. A modified Poisson regression model was used to estimate the prevalence ratio (PR) to account for confounders and multicollinearity, as this approach provides more accurate estimation even for common events (>10 % incidence) [12]. Given the uncertainty regarding optimal events-per-variable (EPV) for modified Poisson regression, we employed multiple regression models to assess the robustness of our findings: Model 1 (BI and UMSARS); Model 2 (Model 1 plus age, sex, and disease duration); and Model 3 (all predefined clinical measures). For subgroup analyses, we employed Model 4, in which the MSA subtype was omitted from Model 3 variables. PRs were calculated per 10-point change in BI (decrease) and UMSARS (increase) for clinical interpretability.

Receiver operating characteristic (ROC) analysis was performed for evaluating discriminative ability. The area under the ROC curve (AUC) assessed overall discriminative ability. The optimal cut-off point was estimated based on the Youden index. Sensitivity and specificity were calculated at the determined cut-off points. All statistical analyses were performed using R software (version 4.3.1; R Foundation, Vienna, Austria). Modified Poisson regression analysis was performed using the rqlm package for R [13].

## 3 Results

### 3.1 Patient selection and demographics

Of 293 patients with MSA who underwent VF, 180 underwent DaT imaging within 12 months. After excluding 75 patients (Supplementary Fig. 1), 105 patients met the eligibility criteria (63 MSA-C, 42 MSA-P; 89 clinically established, 16 clinically probable MSA). We excluded 72 patients lacking UMSARS evaluations due to variations in clinical practice. The study population was 55 % female (58 patients) with a median age of 63.0 years (range, 43–87 years), disease duration of 4.0 years (range, 1–10 years), Barthel index (BI) of 75.0 (range, 0–100), and UMSARS score of 47.0 (range, 18–89). Missing data were found for the MMSE score in five patients (4.8 %).

### 3.2 Group comparison between aspiration and non-aspiration groups

Twenty-eight patients (26.7 %) showed aspiration on VF. After Bonferroni correction, the aspiration group had significantly higher UMSARS (median 59.0 [IQR 48.0–68.3] versus 41.0 [30.0–57.0], p = 0.002) and lower BI (42.5 [30.0–51.3] versus 85.0 [55.0–95.0], p < 0.001) compared with the non-aspiration group (Table 1). There were no significant intergroup differences in terms of age, sex, BMI, MSA subtype, disease duration, MMSE scores, or SBR*mean*. In subtype analysis (Supplementary Table 1), only the BI in the MSA-P group showed a significant intergroup difference (40.0 [25.0–50.0] versus 85.0 [57.5–90.0], p = 0.004).

**Table 1:** Demographic and clinical characteristics of patients with multiple system atrophy (MSA) with and without aspiration.

	Total (n = 105)						
	Aspiration (n = 28)	Non-aspiration (n = 77)	*p*-value (uncorrected)			*p*-value (corected)	
Age, years	65.0 (57.0–72.3)	63.0 (58.0–70.0)		0.528			1.000
Sex (female)	16/28 (57.1 %)	42/77 (54.6 %)		0.829			1.000
BMI	20.1 (17.8–23.5)	21.9 (20.0–25.4)		0.076			0.684
Subtype (MSA-P)	12/28 (42.9 %)	30/77 (39.0 %)		0.823			1.000
Duration, years	5.0 (3.8–5.0)	3.0 (2.0–6.0)		0.129			1.000
MMSE	26.5 (23.8–29.0)	27.0 (25.0–29.0)		0.123			1.000
Barthel index	42.5 (30.0–51.3)	85.0 (55.0–95.0)	<	0.001 **			0.001 **
UMSARS total	59.0 (48.0–68.3)	41.0 (30.0–57.0)	<	0.001 **			0.002 *
SBR*mean*	3.60 (2.34–4.56)	4.68 (2.83–5.64)		0.033 *			0.297

Continuous variables are shown as medians (interquartile ranges) and categorical variables as counts (percentages). P-values were calculated using two-tailed Mann–Whitney *U* test for continuous variables and two-tailed Fisher’s exact test for categorical variables. Corrected p-values were calculated using the Bonferroni correction for nine multiple comparisons; corrected p-values > 1 were truncated to 1.000. * p < 0.05, ** p < 0.01. MSA, multiple system atrophy; MSA-P, MSA parkinsonian type; BMI, body mass index; MMSE, Mini-Mental State Examination; UMSARS, Unified MSA Rating Scale; SBR*mean*, mean specific binding ratio.

### 3.3 Association of clinical measures for aspiration

**Fig. 1:**
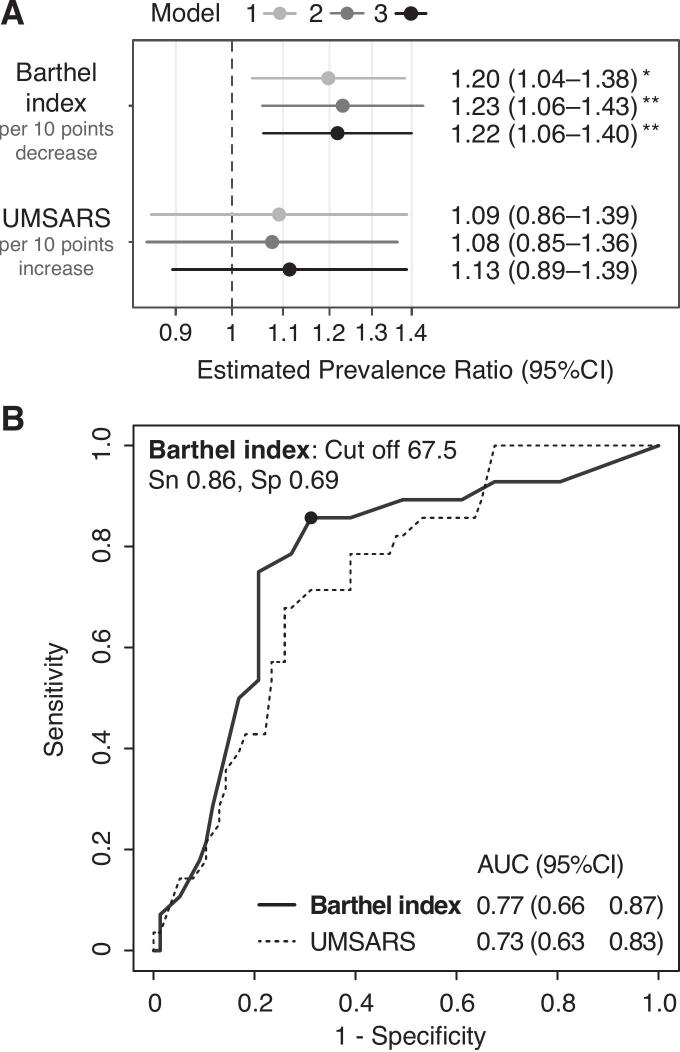
Association and discriminative ability of clinical measures for aspiration risk in MSA. (A). Forest plot showing estimated prevalence ratios (PRs) with 95 % confidence intervals (CIs) for aspiration, visualized on a logarithmic scale. Model 1 included BI and UMSARS; Model 2 added age, sex, and disease duration to Model 1; Model 3 added body mass index, Mini-Mental State Examination score, MSA subtype, and mean specific binding ratio to Model 2. PRs were calculated per 10-point decrease in BI and per 10-point increase in UMSARS. p < 0.05, *p < 0.01. Multiple regression models were employed to assess the robustness of findings. (B). Receiver operating characteristic (ROC) curves showing the discriminative ability of BI and UMSARS for aspiration. The optimal cutoff for BI (67.5) is indicated with corresponding sensitivity and specificity values. AUC: area under the curve; BI: Barthel index; CI: confidence interval; MSA: multiple system atrophy; Sn: sensitivity; Sp: specificity; UMSARS: Unified Multiple System Atrophy Rating Scale.

Initial correlation analysis revealed a strong negative correlation between BI and UMSARS (r = -0.72) and between SBR*mean* and MSA-P subtype (r = -0.66) (Supplementary Fig. 2). In multivariable models, only BI was consistently and significantly associated with aspiration across all models (estimated PRs = 1.20–1.23 per 10-point decrease in BI, all p < 0.01) (Fig. 1A). UMSARS showed no significant association (estimated PRs = 1.08–1.11, all p > 0.34). In subtype analysis, only BI in the MSA-P group showed a significant association with aspiration in Models 1 and 2 (estimated PRs = 1.31 and 1.29, both p < 0.01) (Supplementary Table 2). The distribution histograms of BI and UMSARS scores between the groups (Supplementary Fig. 3) visually support the trend that patients with MSA with lower BI scores are more prone to aspiration.

### 3.4 Discriminative ability of clinical measures for aspiration Detection

The ROC analysis confirmed that BI has a moderate discriminative ability for aspiration (AUC of 0.77 [95 % CI 0.66–0.87]). The optimal cut-off of BI at 67.5 had a sensitivity of 85.7 % and specificity of 68.8 % (Fig. 1B). UMSARS showed relatively weak discriminative ability (AUC 0.73 [0.63–0.83]) (Fig. 1B). Stratified analysis of the MSA-P subtype demonstrated higher AUCs in MSA-P (BI: 0.84 [0.72–0.96]; UMSARS: 0.75 [0.59–0.90]) than the MSA-C (BI: 0.72 [0.57–0.87]; UMSARS: 0.71 [0.57–0.84]) (Supplementary Table 3).

## 4 Discussion

### 4.1 Main findings and clinical significance

In this cross-sectional study of 105 patients with MSA, BI remained consistently and significantly associated with aspiration risk across multiple regression models, with an estimated 20–23 % increased prevalence per 10-point decrease. In the ROC analyses, BI demonstrated a moderate discriminative ability, with an AUC of 0.77. With an optimal cutoff of 67.5, achieving 85.7 % sensitivity, BI shows potential as a clinical marker for aspiration risk in patients with MSA. While UMSARS did not demonstrate consistent statistical significance in regression models, the discriminative ability was comparable (AUC of 0.73), with overlapping confidence intervals. Notably, the association of BI was more pronounced in MSA-P, with both higher effect size and discriminative ability compared with MSA-C.

### 4.2 Interpretation of the results

Both BI and UMSARS demonstrated consistent effect directions in regression models, with BI achieving statistical significance while UMSARS did not. This difference may partly reflect statistical considerations, particularly given the observed multicollinearity between the measures (r = −0.72). ROC analysis revealed similar discriminative abilities with overlapping confidence intervals, indicating the clinical difference is small and uncertain. The cross-sectional nature warrants cautious interpretation of causality. BI's stronger association may reflect better sensitivity to general debilitation from aspiration than UMSARS's disease-specific assessment. We emphasize BI as a practical clinical indicator based on its consistent statistical significance, rather than claiming substantial superiority over UMSARS.

The stronger association with BI in patients with MSA-P compared to MSA-C was observed in our cohort, though inference of biological mechanisms remains uncertain. A potential explanation may derive from BI's focus on activity-level outcomes, whose relationship with pathophysiological progression may differ across subtypes. Given the unclear biological basis, cautious interpretation is warranted when generalizing findings.

Regarding DaT imaging, the absence of an association with aspiration risk contrasts with Parkinson's disease, where reduced SBR is specifically associated with aspiration risk [11]. Although Wada et al. demonstrated correlation between comprehensive swallowing function (Hyodo score) and SBR in patients with MSA [7], our aspiration-focused findings revealed no significant correlations. This discrepancy suggests that distinct pathophysiological mechanisms underlie aspiration and general swallowing dysfunction in patients with MSA.

The absence of an association between disease duration and aspiration risk likely reflects cohort characteristics rather than biological factors in this rapidly progressive disorder. In our cohort, disease duration showed a clustered distribution (IQR 3–6 years), limiting variability for detecting correlations with aspiration. This distribution pattern reflects the typical patient population seen in clinical practice, thus the results are of clinical relevance.

### 4.3 Clinical applications

Our findings have practical implications. BI offers practical advantages for routine monitoring: it can be assessed by caregivers or healthcare providers, in contrast to UMSARS or DaT imaging, which require expert neurologist evaluation or specialized facilities. Incorporating regular BI assessments offers potential benefits: when BI declines, considering that each 10-point decrease is associated with an estimated 20–23 % increased aspiration prevalence on VF in our cohort, clinicians should consider proactive referral for detailed swallowing evaluations. The cutoff of 67.5 (sensitivity, 86 %; specificity, 69 %) could guide decisions even without obvious dysphagia symptoms. BI's discriminative ability appears more reliable in patients with MSA-P, whereas patients with MSA-C may require more careful monitoring regardless of their BI scores.

## 5 Limitations

Some methodological limitations should be noted. The cross-sectional design limits causal inferences about BI’s association with aspiration and prevents evaluation of the temporal relationship between BI decline and the onset of aspiration. Our single-center design, as well as the exclusion of 72 patients due to incomplete clinical assessments, had potential selection bias, limiting its generalizability. Information on L-DOPA treatment in in patients with MSA-P was not systematically collected, which may have influenced the interpretation on observed associations, though recent work shows no group-level effect on aspiration in patients with MSA [14]. The study also lacked data on potentially important comorbidities, including sarcopenia and respiratory function, which may influence aspiration risk. Finally, we focused on basic clinical measures and did not assess comprehensive details, such as motor progression patterns, speech characteristics, postural abnormalities, or non-motor scales, that might provide additional mechanistic insights into aspiration risk. Future research incorporating detailed assessments including individual BI components, brain MRI findings typical for MSA, longitudinal data, and multicenter cohorts is warranted to address these aspects.

## 6 Conclusion

This study suggests BI as a clinical marker of aspiration risk in patients with MSA, particularly promising for those with MSA-P. A decline in BI could indicate the need for swallowing assessments even in the absence of overt dysphagia symptoms. Although the cross-sectional design prevents establishing causality, BI’s simplicity and availability make it an attractive clinical marker. Though prospective validation is needed, implementing BI evaluation could help optimize timing of swallowing assessments in clinical practice.

## CRediT authorship contribution statement

**Jun Tanimura:** Writing – review & editing, Writing – original draft, Visualization, Validation, Software, Methodology, Investigation, Formal analysis, Data curation, Conceptualization. **Toshiyuki Yamamoto:** Writing – review & editing, Resources, Project administration, Methodology, Investigation, Funding acquisition, Conceptualization. **Yuji Takahashi:** Writing – review & editing, Supervision, Project administration.

## Declaration of competing interest

The authors declare that they have no known competing financial interests or personal relationships that could have appeared to influence the work reported in this paper.
