# Supplementary material for: Clinical measures associated with aspiration risk in multiple system atrophy: a cross-sectional study
Source: Clin Park Relat Disord. 2025 Oct 17;13:100401. doi: 10.1016/j.prdoa.2025.100401 (PMC12590134; doi:10.1016/j.prdoa.2025.100401)
Supplement: Supplementary Data 1 [file mmc1.docx]

**Supplementary Table 1**. Subtype analysis of demographic and clinical characteristics in patients with MSA with and without aspiration on VF

|  | **MSA-C** (n=63) | | | |  | **MSA-P** (n=42) | | | |
| --- | --- | --- | --- | --- | --- | --- | --- | --- | --- |
|  | **Aspiration** (n=16) | **Non-aspiration** (n=47) | ***p-*value** (uncorrected) | ***p*-value** (corrected) |  | **Aspiration** (n=12) | **Non-aspiration** (n=30) | ***p-*value** (uncorrected) | ***p*-value** (corrected) |
| Age, years | 66.0 (60.3–71.3) | 61.0 (57.0–66.0) | 0.100 | 0.800 |  | 63.0 (56.5–73.0) | 68.0 (59.5–72.8) | 0.435 | 1.000 |
| Sex (female) | 6/16 (37.5%) | 21/47 (44.7%) | 0.772 | 1.000 |  | 10/12 (83.3%) | 21/30 (70.0%) | 0.464 | 1.000 |
| BMI | 22.4 (19.8–26.2) | 22.3 (20.9–25.7) | 0.801 | 1.000 |  | 17.9 (16.3–19.5) | 21.0 (19.2–24.9) | 0.017 * | 0.138 |
| Duration, years | 4.5 (3.8–6.0) | 3.0 (2.0–6.0) | 0.159 | 1.000 |  | 5.0 (3.8–5.0) | 4.0 (2.3–6.0) | 0.571 | 1.000 |
| MMSE | 25.5 (23.5–28.0) | 28.0 (26.0–29.3) | 0.015 * | 0.118 |  | 28.5 (23.8–29.0) | 25.0 (25.0–29.0) | 0.822 | 1.000 |
| Barthel index | 47.5 (35.0–57.5) | 80.0 (55.0–97.5) | 0.009 ** | 0.073 |  | 40.0 (20.0–50.0) | 85.0 (57.5–90.0) | 0.001 ** | 0.006 * |
| UMSARS total | 54.0 (42.5–64.0) | 41.0 (29.5–51.0) | 0.014 * | 0.112 |  | 64.5 (58.0–71.0) | 41.5 (33.3–62.3) | 0.013 * | 0.105 |
| SBR*mean* | 4.51 (3.71–5.67) | 5.50 (4.77–6.72) | 0.053 | 0.424 |  | 1.94 (1.30–2.93) | 2.80 (2.38–3.54) | 0.055 | 0.438 |

Data are presented separately for the MSA-C and MSA-P subtypes. Continuous variables are shown as medians (interquartile ranges) and categorical variables as counts (percentages). P-values were calculated using two-tailed Mann–Whitney U test for continuous variables and two-tailed Fisher’s exact test for categorical variables. Corrected p-values were calculated using the Bonferroni correction for eight multiple comparisons; corrected p-values > 1 were truncated to 1.000.

* p < 0.05, ** p < 0.01. MSA, multiple system atrophy; MSA-C, MSA cerebellar type; MSA-P, MSA parkinsonian type; BMI, body mass index; MMSE, Mini-Mental State Examination; UMSARS, Unified MSA Rating Scale; SBR*mean*, mean specific binding ratio.
