# Supplementary material for: Clinical measures associated with aspiration risk in multiple system atrophy: a cross-sectional study
Source: Clin Park Relat Disord. 2025 Oct 17;13:100401. doi: 10.1016/j.prdoa.2025.100401 (PMC12590134; doi:10.1016/j.prdoa.2025.100401)
Supplement: Supplementary Data 2 [file mmc2.docx]

**Supplementary Table 2**. Association between the Barthel index, UMSARS scores, and aspiration in patients with MSA, analyzed by modified Poisson regression analysis

|  | **Model 1** |  |  | **Model 2** |  |  | **Model 3** |  |
| --- | --- | --- | --- | --- | --- | --- | --- | --- |
|  | estimated PR (95%CI) | *p*-value |  | estimated PR (95%CI) | *p*-value |  | estimated PR (95%CI) | *p*-value |
| **MSA all** |  |  |  |  |  |  |  |  |
| Barthel index (10-point decrease) | 1.20 (1.04―1.38) | 0.01 * |  | 1.23 (1.06―1.43) | < 0.01 ** |  | 1.22 (1.06―1.40) | < 0.01 ** |
| UMSARS (10-point increase) | 1.09 (0.86―1.39) | 0.47 |  | 1.08 (0.85―1.36) | 0.53 |  | 1.11 (0.89―1.39) | 0.34 |
|  |  |  |  |  |  |  |  |  |
|  | **Model 1** |  |  | **Model 2** |  |  | **Model 4** |  |
|  | estimated PR (95%CI) | *p*-value |  | estimated PR (95%CI) | *p*-value |  | estimated PR (95%CI) | *p*-value |
| **MSA-C** |  |  |  |  |  |  |  |  |
| Barthel index (10-point decrease) | 1.14 (0.95―1.37) | 0.15 |  | 1.19 (0.98―1.45) | 0.08 |  | 1.16 (0.96―1.40) | 0.14 |
| UMSARS (10-point increase) | 1.15 (0.83―1.59) | 0.40 |  | 1.07 (0.77―1.49) | 0.69 |  | 1.09 (0.79―1.51) | 0.60 |
| **MSA-P** |  |  |  |  |  |  |  |  |
| Barthel index (10-point decrease) | 1.31 (1.11―1.56) | < 0.01 ** |  | 1.29 (1.09―1.53) | < 0.01 ** |  | 1.29 (0.95―1.76) | 0.11 |
| UMSARS (10-point increase) | 0.98 (0.71―1.38) | 0.95 |  | 1.11 (0.75―1.63) | 0.60 |  | 1.30 (0.80―2.10) | 0.29 |

Results are presented as estimated prevalence ratios (PRs) with 95% confidence intervals (CIs). The PRs were calculated for a 10-point decrease in the BI and a 10-point increase in the UMSARS. Model specifications: Model 1 (adjusted for BI and UMSARS), Model 2 (Model 1 + age, sex, and disease duration), Model 3 (Model 2 + BMI, MMSE, MSA subtype, and SBR*mean*), and Model 4 (Model 2 + BMI, MMSE, and SBR*mean*). Model 3 was used for all patients with MSA and Model 4 was used for subtype analyses. We employed multiple regression models for assessing the robustness of findings.

* p < 0.05, ** p < 0.01. MSA, multiple system atrophy; MSA-C, MSA cerebellar type; MSA-P, MSA Parkinsonian type; BI, Barthel index; UMSARS, Unified MSA Rating Scale; BMI, body mass index; MMSE, Mini-Mental State Examination; SBR*mean*, mean specific binding ratio.
