# Supplementary material for: Clinical measures associated with aspiration risk in multiple system atrophy: a cross-sectional study
Source: Clin Park Relat Disord. 2025 Oct 17;13:100401. doi: 10.1016/j.prdoa.2025.100401 (PMC12590134; doi:10.1016/j.prdoa.2025.100401)
Supplement: Supplementary Data 3 [file mmc3.docx]

**Supplementary Table 3.** Discriminative ability of clinical measures for aspiration in MSA evaluated by ROC analysis

|  | AUC (95%CI) | Cut-off | Sensitivity (%) | Specificity (%) |
| --- | --- | --- | --- | --- |
| **All** |  |  |  |  |
| Barthel index | 0.77 (0.66―0.87) | 67.5 | 85.7 | 68.8 |
| UMSARS | 0.73 (0.63―0.83) | 54.5 | 67.9 | 74.0 |
| **MSA-C** |  |  |  |  |
| Barthel index | 0.72 (0.57―0.87) | 67.5 | 81.2 | 70.2 |
| UMSARS | 0.71 (0.57―0.84) | 33.5 | 100.0 | 36.2 |
| **MSA-P** |  |  |  |  |
| Barthel index | 0.84 (0.72―0.96) | 52.5 | 83.3 | 80.0 |
| UMSARS | 0.75 (0.59―0.90) | 54.0 | 91.7 | 60.0 |

The results are shown as the area under the receiver operating characteristic curve (AUC) with 95% confidence intervals (CIs). The optimal cutoff points were determined using the Youden index, with corresponding sensitivity and specificity values. MSA, multiple system atrophy; MSA-C, MSA cerebellar type; MSA-P, MSA Parkinsonian type; ROC, receiver operating characteristic; UMSARS: Unified MSA Rating Scale; SBR*mean*, mean specific binding ratio.
