# Supplementary figures and images for: Clinical measures associated with aspiration risk in multiple system atrophy: a cross-sectional study
Source: Clin Park Relat Disord. 2025 Oct 17;13:100401. doi: 10.1016/j.prdoa.2025.100401 (PMC12590134; doi:10.1016/j.prdoa.2025.100401)

**A**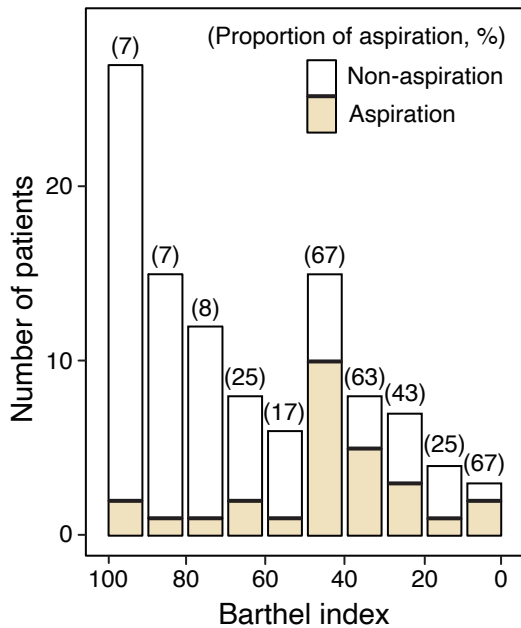**B**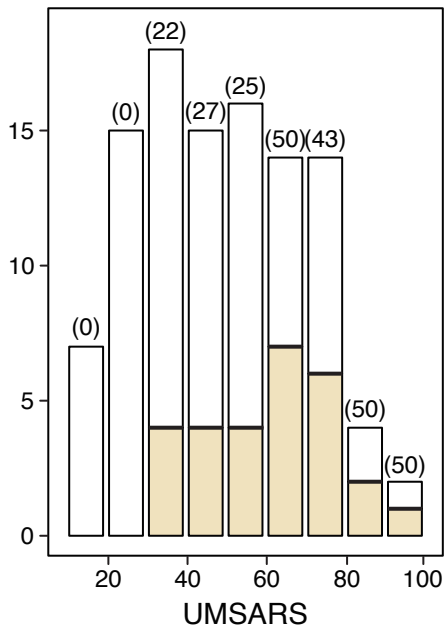

Supplement: Supplementary Data 4 [file mmc4.pdf]

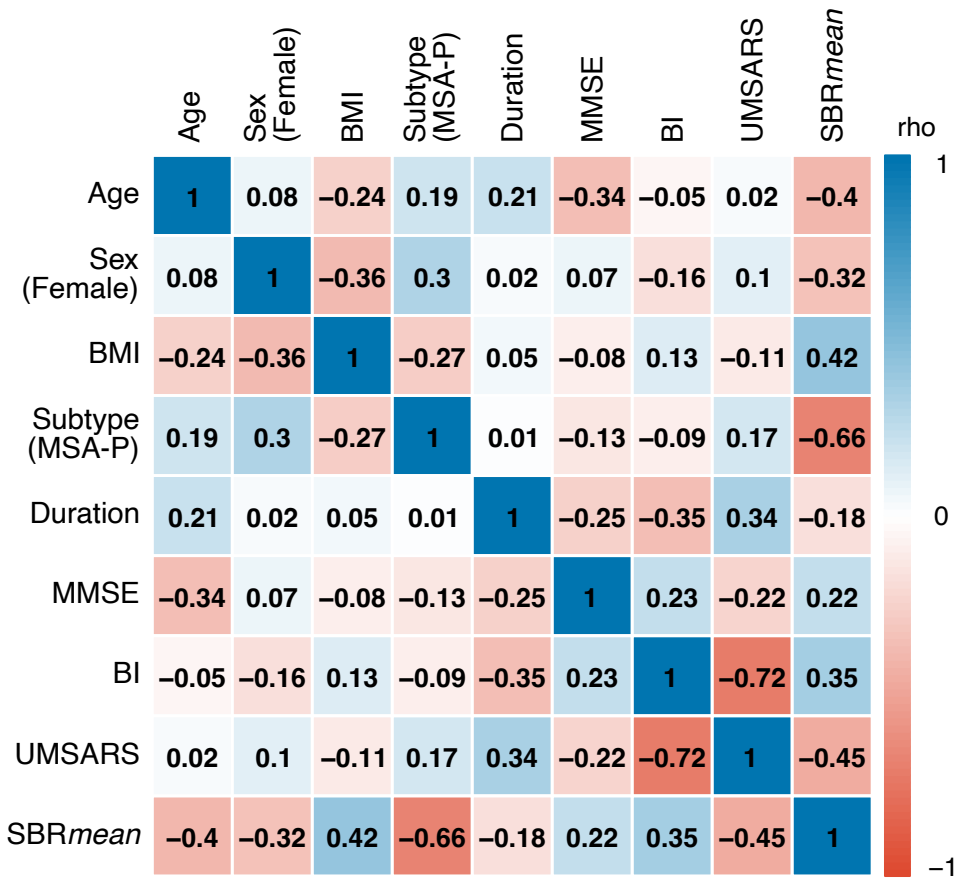

Supplement: Supplementary Data 6 [file mmc6.pdf]
