# Supplementary material for: Clinical measures associated with aspiration risk in multiple system atrophy: a cross-sectional study
Source: Clin Park Relat Disord. 2025 Oct 17;13:100401. doi: 10.1016/j.prdoa.2025.100401 (PMC12590134; doi:10.1016/j.prdoa.2025.100401)
Supplement: Supplementary Data 5 [file mmc5.pdf]

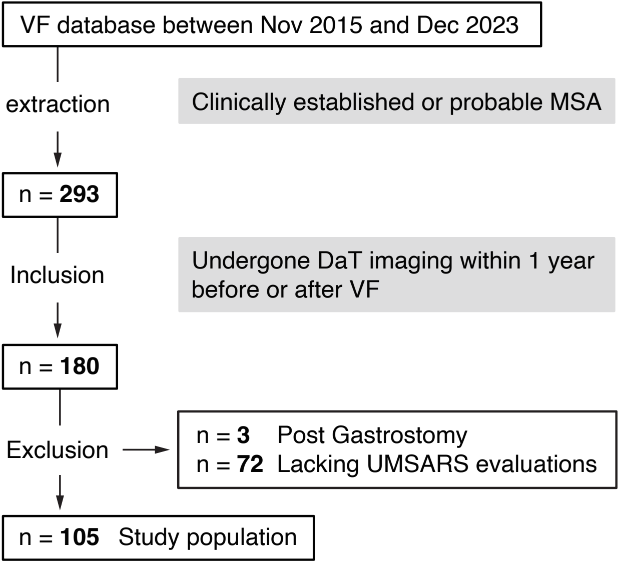
VF database between Nov 2015 and Dec 2023
extraction
Clinically established or probable MSA
n = 293
Inclusion
Undergone DaT imaging within 1 year before or after VF
n = 180
Exclusion
n = 3 Post Gastrostomy
n = 72 Lacking UMSARS evaluations
n = 105 Study population
